# Hippocampal responses to electrical stimulation of the major input pathways are modulated by dentate spikes

**DOI:** 10.1002/hipo.23470

**Published:** 2022-09-16

**Authors:** Suvi‐Maaria Lehtonen, Tomi Waselius, Markku Penttonen, Miriam S. Nokia

**Affiliations:** ^1^ Department of Psychology University of Jyvaskyla Jyvaskyla Finland

**Keywords:** dentate gyrus, electrophysiology, hippocampus, lateralization, synaptic transmission

## Abstract

Dentate gyrus (DG) is important for pattern separation and spatial memory, and it is thought to gate information flow to the downstream hippocampal subregions. Dentate spikes (DSs) are high‐amplitude, fast, positive local‐field potential events taking place in the DG during immobility and sleep, and they have been connected to memory consolidation in rodents. DSs are a result of signaling from the entorhinal cortex (EC) to the DG, and they suppress firing of pyramidal cells in the CA3 and CA1. To study the effects of DSs to signaling in the hippocampal tri‐synaptic loop, we electrically stimulated the afferent fibers of the DG, CA3, and CA1 in adult male Sprague–Dawley rats at different delays from DSs. Responses to stimulation were increased in the EC‐DG synapse during DSs, and the effect was amplified after theta‐burst stimulation. We concluded that DSs strengthen the excitatory signal from the EC to the DG, which is reinforced by synapse potentiation and increased excitability of granule cells after theta‐burst stimulation. This signal boosting may function in enhancing plastic changes in the DG‐CA3 synapse. As responses in the CA3 and CA1 remained unaffected by the DS, the DS‐contingent silencing of pyramidal cells seems to be a result of a decrease in excitatory input rather than a decrease in the excitability of the pyramidal cells themselves. In addition, we found that the DSs occur asynchronously in the left and right hippocampi, giving novel evidence of lateralization of the rodent hippocampus.

## 1 INTRODUCTION

The hippocampus is widely accepted to be important for memory, and the structure of the hippocampal “tri‐synaptic loop” is well documented (Amaral & Witter, 1989). The neural circuitry of the dentate gyrus (DG) consists of excitatory input from the entorhinal cortex (EC) to the DG granule cells, mossy cells and GABAergic hilar interneurons, the local synaptic interactions of these cells, and their output to the CA3 (Bragin et al., 1995; Penttonen et al., 1997; Senzai & Buzsáki, 2017; Sik et al., 1997). Interestingly, DG granule cells seem to innervate more inhibitory interneurons than excitatory CA3 pyramidal cells (Acsády et al., 1998). According to a conductance‐based analysis by Treviño et al. (2011) when DG granule cells fire action potentials, connected interneuron membrane potentials become more depolarized than those of CA3 pyramidal cells. This suggests that the effects of granule cell firing on CA3 pyramidal cells should be understood as a balance of feedforward excitation and inhibition, which can be shifted by altering granule cell firing rate (Henze et al., 2002; Mori et al., 2004).

In awake state, DG granule cells fire rarely (Pofahl et al., 2021; Senzai & Buzsáki, 2017) and are thought to function as filters for information transmission from the EC to CA3 pyramidal cells. The sparse firing in DG is suggested to indicate a role in pattern separation and in the formation of spatial memories (Leutgeb et al., 2007; O'Reilly & McClelland, 1994; Pofahl et al., 2021). However, during rest and especially non‐rapid eye movement (NREM) sleep, when memory consolidation is thought to take place (Buzsaki, 2002, 2015), irregular bursting activity takes place in the DG: so‐called dentate spikes (DSs) arise when excitatory EC input causes strong synchronous activation of mostly granule cells, but also mossy cells and interneurons (Bragin et al., 1995; Penttonen et al., 1997; Senzai & Buzsáki, 2017). DSs can be detected in the hilar local‐field potentials (LFPs) as high amplitude (1–4 mV), brief (10–40 ms) spikes of positive potential (Bragin et al., 1995; Penttonen et al., 1997), and they are common especially during neocortical up‐states (Headley et al., 2017).

Whereas the role of CA1 sharp‐wave ripples (SPW‐Rs) in memory consolidation is widely accepted (Buzsaki, 2015; Girardeau et al., 2009; Wilson & McNaughton, 1994), less is known about the meaning of DSs. DSs inhibit CA3 and CA1 firing, possibly because of the connections from granule cells to GABAergic interneurons, which connect to CA3 pyramidal cells in feed‐forward manner. During DSs, SPW‐Rs occur less frequently for a period of up to 200 ms (Bragin et al., 1995; Headley et al., 2017; Penttonen et al., 1997). Further, DSs seem to take place more likely right after SPW‐Rs (Headley et al., 2017). This interaction between SPW‐Rs and DSs suggests that DSs are capable of modulating memory consolidation in the CA1, and in fact recent studies indicate that disrupting the naturally occurring DSs during a rest period immediately after training affects performance in hippocampus‐dependent learning tasks (Lensu et al., 2019; Nokia et al., 2017). Why this happens and what are the effects of DSs on signal flow between the hippocampal subregions are not completely understood.

We investigated the effects of DSs on synaptic transmission in the hippocampal tri‐synaptic loop in urethane anesthetized adult male Sprague Dawley rats. We stimulated the perforant path (PP), mossy fibers (MF), and the ventral hippocampal commissure (vHC) electrically at different timepoints (0–30, 31–100, 400–600, >1000 ms) after DSs. Simultaneously, we recorded LFPs in the DG, CA3, and CA1. To see if the effect is different after synapse potentiation, we recorded responses both prior to and after theta‐burst stimulation. Previously it has been reported that stimulation of the medial PP during DSs in immobile alert wake rats leads to enhanced granule cell excitation (Bramham, 1998), possibly maximizing the signal transmission between the EC and DG. However, to our knowledge no research has been conducted in other synapses of the tri‐synaptic loop. In line with Bramham (1998), we expected that during and immediately after DSs response amplitude in the DG increases. In contrast, when stimulating MFs and vHC during DSs, a decrease or no effect in the amplitude should be seen in the CA3 and CA1.

## 2 MATERIAL AND METHODS

### 2.1 Ethics statement

All the experimental procedures were approved by the Animal Experiment Board of Finland and implemented in accordance with directive 2010/63/EU of the European Parliament and of the Council on the care and use of animals for research purposes.

### 2.2 Animals

Adult male Sprague–Dawley rats purchased from Envigo (Netherlands) were used as subjects. The rats were housed at the Laboratory center of the University of Jyväskylä in groups (Macrolon IV, Techniplast, Italy) until the surgery. Aspen chips (Tapvei, Estonia) were used as bedding and a plastic toy was provided to each cage. Housing conditions were controlled with temperature at 21 ± 2°C, and humidity at 50 ± 10%. The rats were kept in 12‐h light–dark cycle, with lights on from 8.00 a.m. Food (R36; Labfor, Lantmännen, Stockholm, Sweden) and water were available ad libitum. The studies were conducted during the light portion of the day. The rats weighed at least 280 g at the time of the surgery.

### 2.3 Surgery

The animals were weighed and then anesthetized with urethane (≥99%, Sigma‐Aldrich, diluted in sterile water at 0.24 g/ml; dose: 1.3–1.5 g/kg administered i.p.). The head of the animal was attached to a stereotaxic instrument once the rat did not react to pinching of the toe or tail. Under local analgesia (bupivacaine 2.5 mg/ml, 0.8 ml s.c.), the skin was removed, and craniotomies made above the left hippocampus and the contralateral vHC, the ipsilateral angular bundle of the PP, or the posterior dorsal hippocampus. A 27 G injection needle was inserted to the cerebellum to serve as the reference and another needle was inserted to the neck muscle to serve as ground. LFPs were recorded with 32‐ch linear silicon probes (Atlas Neuroengineering, Belgium, catalog nr. E32‐65‐S1‐L6‐NT). The stimulation electrode was made of Formwar‐coated stainless‐steel dual‐wire with a diameter of 100 μm and a tip separation of ~500 μm.

**FIGURE 1:**
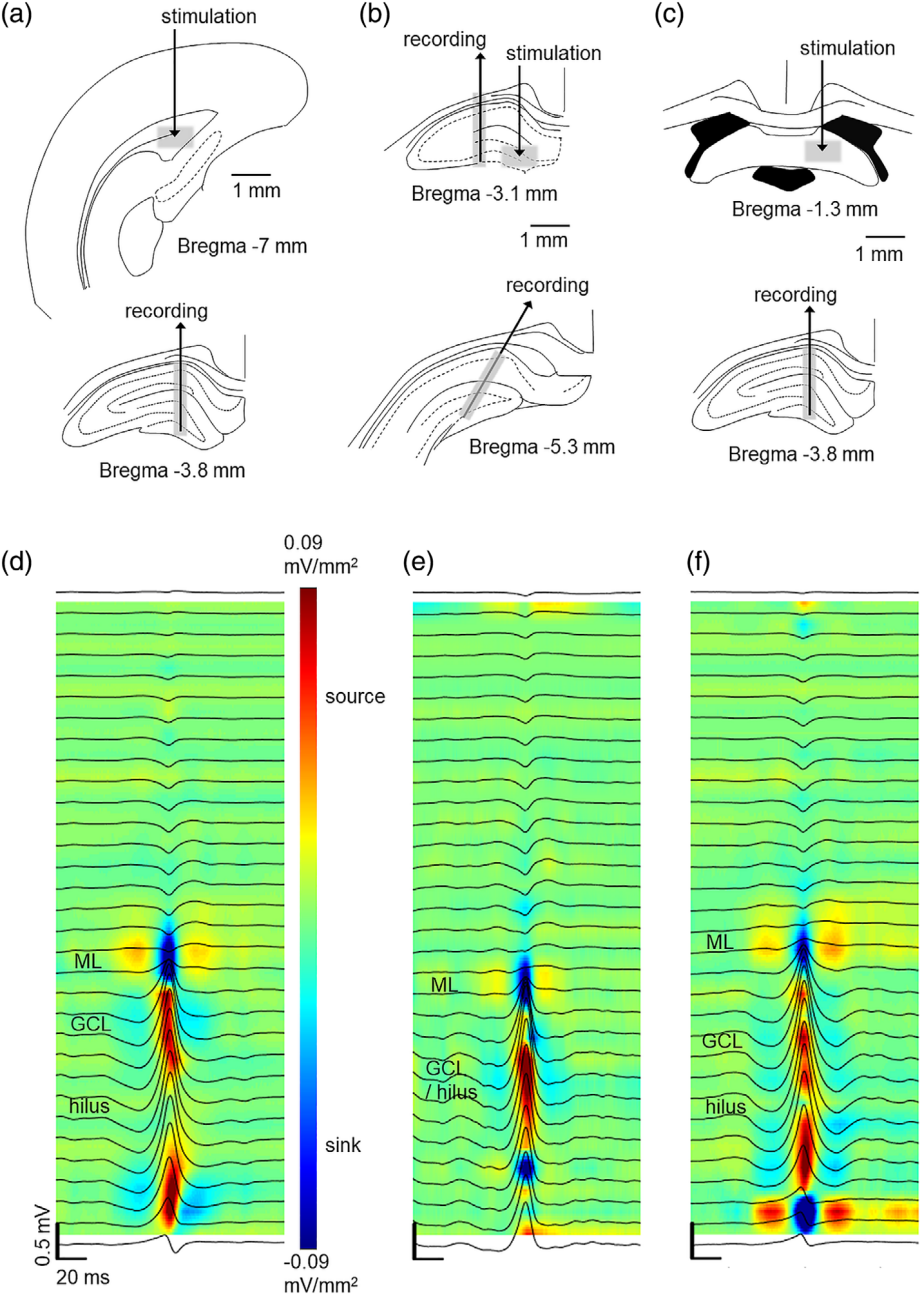
The effect of dentate spikes on responses to electrical stimulation was studied in the entorhinal cortex to the dentate gyrus (EC‐DG) synapse (a), the DG‐CA3 synapse (b) and the CA3‐CA1 synapse (c) in adult male Sprague–Dawley rats under urethane anesthesia. Approximate stimulation and recording sites are illustrated with gray shading. (d, e, and f) panels illustrate the current source density map (blue = sink, red = source) and average local‐field potential traces during dentate spikes recorded from representative rats in each respective experiment. Note that the data for (d–f) are from the 1‐h baseline recording during which no stimulations were administered. A sink is visible in the molecular layer and source in the DG during dentate spikes

Electrode locations are illustrated in Figure 1. First, to replicate earlier findings (Bragin et al., 1995) regarding bilateral synchrony of DSs, we implanted linear probes to dorsal hippocampi in the left and the right hemisphere at symmetrical positions (3.6–4.0 mm posterior (P), 2 mm lateral (L) to bregma) in three rats. To also verify the longitudinal synchrony of DSs (Bragin et al., 1995), in the same three rats, we then implanted linear probes to the anterior (P 2.8–3 mm, L 1.5–2 mm) and posterior (P 5.0–5.5, L 2.5–3.0 mm) dorsal hippocampus in the left hemisphere. In separate rats, to study synaptic transmission from the EC to the DG, a linear probe was implanted to the dorsal hippocampus (P 3.8 mm, L 1.7 mm), targeting the tip at the lower blade of the DG (~3.6 mm below dura) and the bipolar stimulation electrode was inserted into the ipsilateral angular bundle of the PP (P 7 mm, L 4 mm) at ~4 mm below dura. To study synaptic transmission from the DG to the CA3, a linear probe was targeted to the CA3 (P 3 mm, L 2.5–3.0 mm) and a stimulation electrode was targeted to the ipsilateral MFs (P 3.0–3.6 mm, L 1.5 mm) at ~3.7 mm below dura. Additionally, to be able to record the DSs, another linear probe was implanted to the ipsilateral posterior dorsal hippocampus at an angle of 30° (P 5.0–6.0 mm, L 2.5–3.0 mm). Finally, to study synaptic transmission from the CA3 to the CA1, a linear probe was implanted to the dorsal hippocampus (P 3.8 mm, L 1.7 mm) and the stimulation electrode was implanted to the contralateral vHC (P 1 mm, L 1 mm) at ~3.5 mm below dura. To be able to fit both stimulation and recording electrodes to the area, vHC was used for stimulation instead of Shaffer collaterals (Bliss et al., 1983).

### 2.4 Recording and stimulation

All stimulations were delivered using an 8‐channel stimulator (STG4008‐160 μA, MultiChannel Systems [MCS], Germany) and controlled using MC_Stimulus II software (MCS) and LabVIEW (National Instruments Corporation, Austin, TX, USA). To record LFPs, a low‐noise pre‐amplifier (4×, μPA32, MCS) was directly attached to the probe connector and the signal conveyed via cable to the filter‐amplifier (FA64I, 0.1–5000 Hz, 50×, MCS). All signals were digitized (USB‐ME‐64 System, MCS), recorded and stored with Mc_Rack software (MCS) using a 20‐kHz sampling rate.

First, the proper stimulation current was determined by applying a single bipolar pulse of 200 μs in total duration every 5 s. The current of the pulse varied from 20 to 160 μA, in 20 μA steps. A total of 20 pulses per current were applied. Then, a 1‐h baseline recording without any stimulation was started. While recording baseline activity, the responses to the stimulation were analyzed offline with MATLAB (The MathWorks Inc., Natick, MA, USA) to determine the current needed to elicit a population spike and a reliable response of ~2 mV at a delay of <10 ms in the target region. Further, during the baseline recording the detection of the DSs was optimized for each animal: To deliver stimulation based on the DSs, signal from a recording electrode on the linear probe showing clear DSs in the hilus was conveyed to LabVIEW. DSs were detected based on a fast rise in signal amplitude and a simple peak amplitude threshold.

After completing the baseline recording, 120 single bipolar stimulation pulses using the chosen current were applied. Stimulations were timed at least 20 s apart, based on detection of a DS in the hilus of the dorsal hippocampus. The stimuli were randomly presented at a delay of 0–30, 31–100, 400–600, or >1000 ms from the DS. After this, theta‐burst stimulation (TBS) routinely used to induce long‐term potentiation was applied at a current needed to induce near‐maximal responses in the target area. TBS stimulation was conducted as follows: Four bipolar 200‐μs pulses were repeated four times at 100 Hz. These bursts were repeated eight times at 5 Hz and this repeated four times at an interval of 20 s (=128 pulses). Next, 120 stimulations at the same lower current used before TBS stimulation were again delivered based on DS detection as described above.

### 2.5 Data analysis

MATLAB was used for off‐line data analysis. First, to verify the stimulation conditions, hippocampal LFPs from each trial were manually checked and the trial assigned to one of the four categories based on the delay from a DS. DSs were detected off‐line as described in Lensu et al. (2019). The acceptable delay ranges used for categorizing were 0–30, 31–100, 400–600, and >1000 ms from DS peak. Trials that did not unambiguously fall into one of the four categories were excluded from further analysis. The resulting number of trials [M(SD)] in each category (0–30, 31–100, 400–600 and >1000 ms) was 19(5), 20(5), 19(4), and 25(7), respectively.

Next, to determine the responses to stimulation, current‐source density (CSD) analysis was performed on the LFPs. The signal corresponding to the maximal sink in the stratum radiatum in CA1 (vHC stimulation), the molecular layer in the DG (PP stimulation) or in the CA3 pyramidal cell layer (MF stimulation) was identified. Maximum sink values were derived from the selected CSD signal during the first 10 ms after stimulation. Then, these values were divided by the mean of the values obtained for all stimulations within a recording (pre‐TBS and post‐TBS) and multiplied by 100. This relative measure was used to study the possible effects that the DS might have on the responses. To probe the effect of TBS overall we also calculated the amplitude of all post‐TBS responses relative to the pre‐TBS responses (change in %). Statistical testing was conducted with IBM SPSS Statistics version 26. Repeated measured analysis of variance (ANOVA) was used to compare relative response amplitudes evoked at the four different delays from DS. Post‐hoc pairwise comparisons were conducted using least significant difference (LSD) correction for *p*‐values. One sample *t*‐test was used to determine the effect of TBS on response amplitudes overall.

### 2.6 Euthanasia and histology

Animals were euthanized with an overdose of pentobarbital (>120 mg/kg, i.p.; Mebunat Vet 60 mg/ml, Orion, Espoo, Finland). Death was verified by decapitation. To verify electrode locations, from the first few animals in each preparation, the brain was extracted and fixed in 4% paraformaldehyde solution (pH 7.4) for at least 48 h at +4°C. The brain was coronally sectioned (40 μm, Leica VT1000), slices were attached to slides, dried, and stained with Prussian blue and Cresyl violet. The electrode tip locations were determined with the help of a conventional light microscope and a brain atlas (Paxinos & Watson, 1998). For the remainder of the animals the electrodes were determined to be in place (or not) based on signal properties, that is, properties of spontaneous oscillations and responses to stimulation.

## 3 RESULTS

### 3.1 Dentate spikes occur simultaneously in anterior and posterior dorsal hippocampus

**FIGURE 2:**
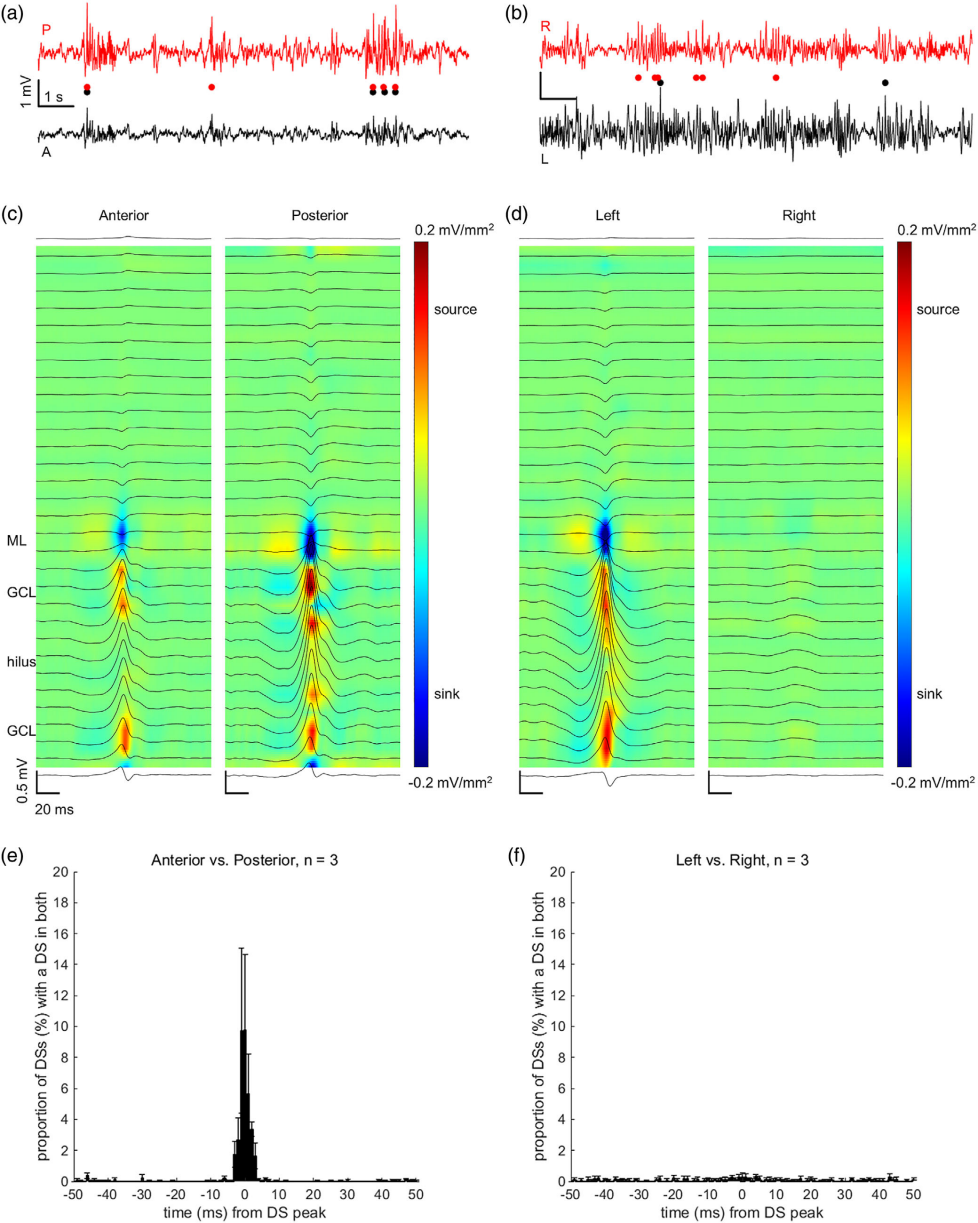
Dentate spikes (DSs) took place simultaneously in both the anterior and the posterior left dorsal hippocampus (a, c, e) but not in the left and right dorsal hippocampus (b, d, f). The timing of DSs within and between hemispheres was studied in three adult male Sprague–Dawley rats under urethane anesthesia. (a) and (b) Example local‐field potentials with DSs from a representative rat. DS timing is marked below with circles. A = anterior, P = posterior, L = left, R = right. (c) Example from the same rat, centered on the peak of the DSs detected in the anterior left dorsal hippocampus. Note that illustrations centered on DSs detected in the posterior dorsal hippocampus indicated DSs also in the anterior dorsal hippocampus. Coloring illustrates the current source density (mV/mm^2^). (d) Example from the same rat, centered on the peak of DSs detected in the left dorsal hippocampus. Note that illustrations centered on DSs detected in the right dorsal hippocampus indicated DSs only in the right dorsal hippocampus. (e, f) Data were averaged per rat and grand averages of the whole group are presented. Vertical lines indicate standard error of mean

We determined whether DSs take place simultaneously (within ±10 ms) in both hippocampi (*n* = 3 rats) as suggested by Bragin et al. (1995). It turned out that this was the case only in a fraction (3.62 ± 1.14%) of the DSs as evident from Figure 2. Next, in the same rats, we determined whether DSs take place simultaneously in the anterior and posterior parts of the dorsal hippocampus. Interestingly, a significant proportion (35.50 ± 15.84%) of DSs were detectable from both the anterior and the posterior linear probe implanted ipsilaterally in the left dorsal hippocampus.

### 3.2 Response to electrical stimulation of the perforant path is stronger in the dentate gyrus during dentate spikes

**FIGURE 3:**
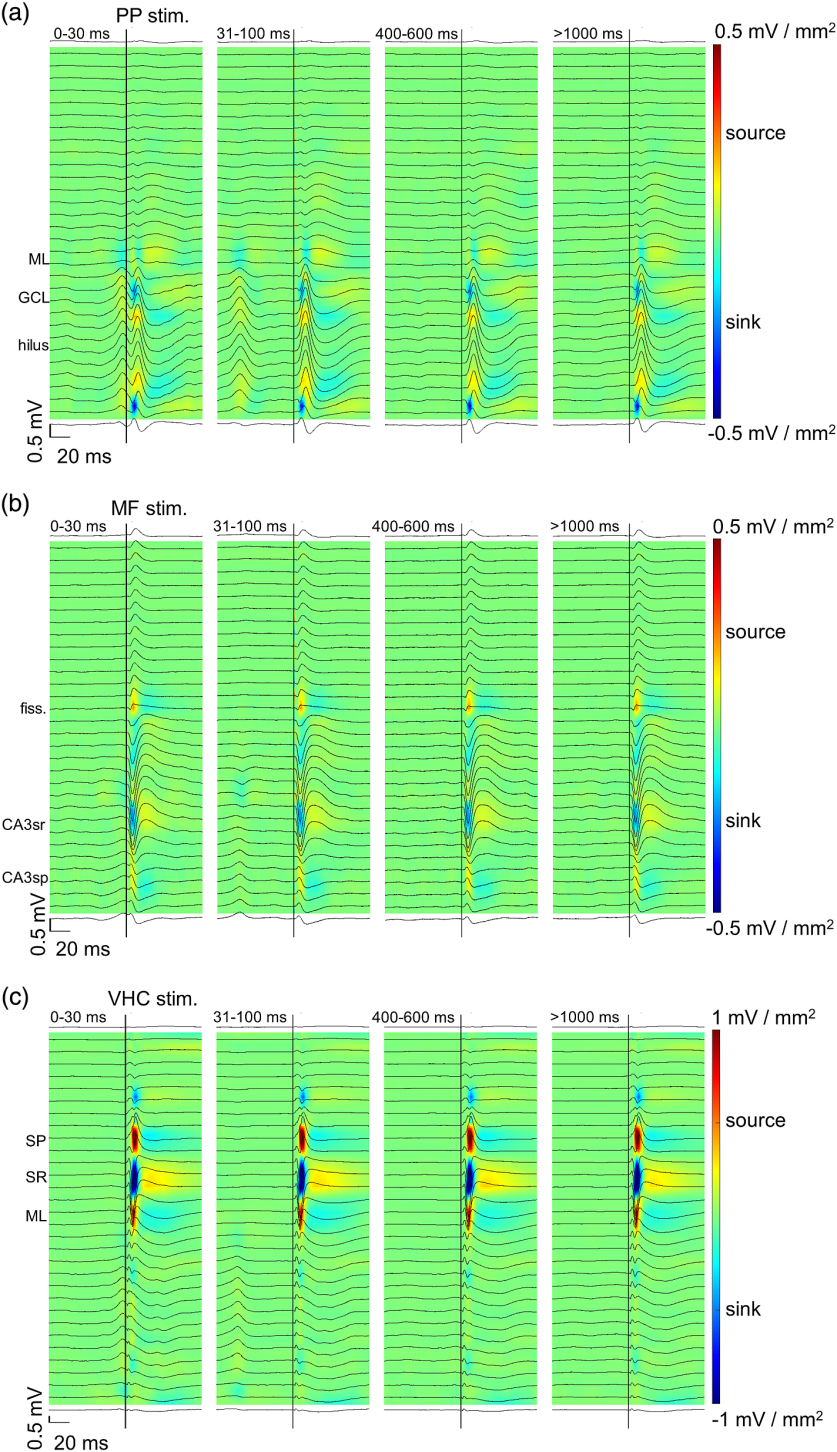
Responses to electrical stimulation in the hippocampal tri‐synaptic loop revealed an effect of the dentate spike on synaptic transmission from the entorhinal cortex to the dentate gyrus (EC‐DG) via the perforant path (PP). (a) Responses to the PP stimulation at different delays from the dentate spike in a representative rat. (b) Responses to the mossy fiber (MF) stimulation at different delays from the dentate spike in a representative rat. (c) Responses to ventral hippocampal commissure (VHC) stimulation at different delays from the dentate spike in a representative rat. Coloring illustrates the current source density map where blue denotes sink and red denotes source. Data from the same rats were used as in Figure 1

**FIGURE 4:**
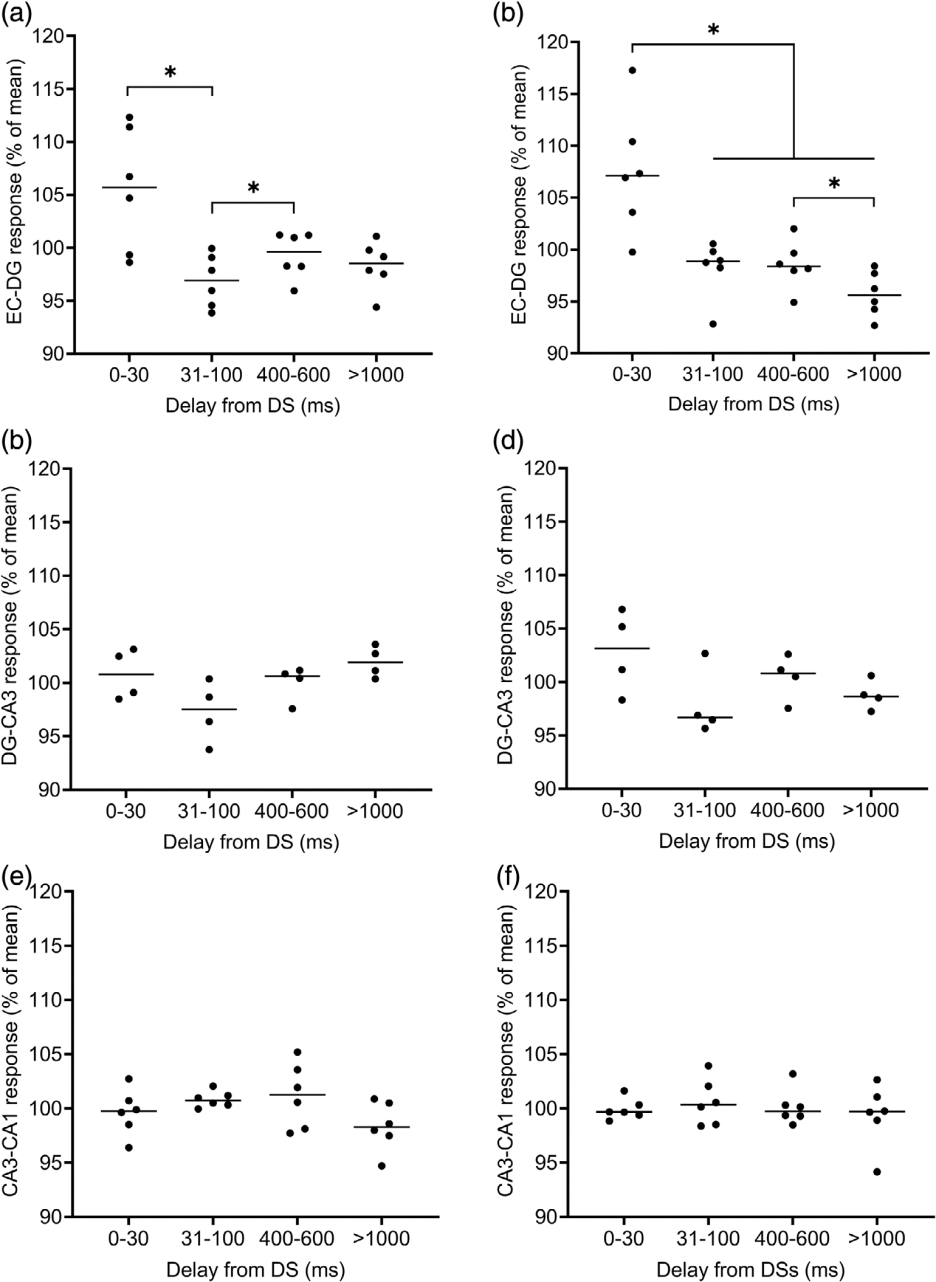
Dentate spike ‐contingent effects of electrical stimulation on synaptic transmission in the hippocampal tri‐synaptic loop. (a) Prior to theta‐burst stimulation, repeated measures ANOVA (least significant difference) revealed dentate spikes (DS)‐related enhancement in the entorhinal cortex to the dentate gyrus (EC‐DG) synapse at delay 0–30 ms from DSs compared to a 31–100 ms delay (*p* = .011). (b) After theta‐burst stimulation, a statistically significant difference was found when 0–30 ms delay was compared to 31–100 (*p* = .043), 400–600 (*p* = .042), and >1000 ms (*p* = .013) delays. In addition, the amplitude at >1000 ms was lower than at 400–600 ms (*p* = .031). (c–f) Results from the CA3 and CA1 synapses prior to (c, e) and after (d, f) theta‐burst stimulation. Values of the Y‐axis are relative local‐field potential sink amplitudes (%) calculated from the mean of all values within a recording

The results are visualized in Figure 3a. A repeated measures ANOVA conducted on data collected from the EC‐DG synapse prior to TBS revealed a statistically significant effect of stimulation delay (*F*[3, 15] = 5.23, *p* = .011). Further analysis with pairwise comparison showed a larger sink in the DG (LSD‐corrected *p* = .047) when the PP stimulation was applied at a delay of 0–30 ms from the DS (mean ± standard deviation: 105.52 ± 5.81%) compared to when it was applied at a 31–100 ms delay (96.88 ± 2.47%). Further, a smaller sink in the DG was detected at a 31–100 ms than at a 400–600 ms delay (99.31 ± 2.17%; *p* = .042). However, there was no difference in the response (*p* = .074) elicited at a 0–30 ms delay from a DS and that elicited by random stimulation at a delay of >1000 ms from a DS (98.30 ± 2.31%) (Figure 4a). A similar effect was evident also after TBS (*F*[1.22, 6.10] = 9.05, *p* = .021) when comparing responses elicited at a delay of 0–30 ms from a DS (107.55 ± 5.99%) to those elicited at a delay of 31–100 ms (98.19 ± 2.75%, *p* = .043), 400–600 ms (98.55 ± 2.32%, *p* = .042), or >1000 ms (95.71 ± 2.17%, *p* = .013) from a DS. In addition, the pairwise comparison revealed a statistically significant difference between responses elicited at delays of 400–600 and >1000 ms (*p* = .031) (Figure 4b). Note that TBS increased the response amplitudes by on average 8.45 ± 19.60% (one sample *t*‐test: *t*(5) = 1.056, *p* = .339). To summarize, DSs modulated the signal transduction from the EC to the DG via the PP: Responses were augmented by the DS and the effect was even more prominent after TBS.

For the DG‐CA3 synaptic transmission, we analyzed the relative amplitude of sinks in the CA3 in response to MF stimulation (Figure 3b). Repeated measures ANOVA indicated no change in response amplitude either prior to TBS (*F*[3, 9] = 2.54, *p* = .122) or after TBS (*F*[3, 9] = 1.80, *p* = .218). TBS itself increased the response amplitudes by only 3.57 ± 23.58% (one sample *t*‐test: *t*[3] = 0.303, *p* = .782). Similar analysis was conducted for the CA3‐CA1 synaptic transmission (Figure 3c). Again, no effect was found either prior to TBS (*F*[3, 15] = 1.56, *p* = .241) or after TBS (*F*[3, 15] = 0.29, *p* = .835). However, TBS was effective in increasing response amplitudes overall by 38.23 ± 34.98% (one sample *t*‐test: *t*(5) = 2.677, *p* = .044). Thus, DSs did not modulate the signal transduction from the DG to the CA3, or from the CA3 to the CA1. The results are illustrated in Figure 4c–f.

## 4 DISCUSSION

In this research, we examined how DSs modulate signal transmission in the hippocampal tri‐synaptic loop in urethane‐anesthetized adult male rats and whether DSs occur synchronously in both hemispheres and in the anterior and posterior parts of the dorsal hippocampus. Contrary to our expectations, DSs were detected simultaneously unilaterally but not bilaterally, as previously suggested (Bragin et al., 1995). Our results thus challenge the existing view of the bilaterality of hippocampal electrophysiological function. Further, our results demonstrate that the effects of DSs on signal transmission within the hippocampus are synapse‐ and timing‐specific: Responses to stimulation were amplified in the DG when stimulation of the PP was conducted during, but not after (>30 ms) a DS, conforming to observations in awake rats by Bramham (1998). As a completely novel finding, we detected no effect of DS on signaling efficiency in the DG‐CA3 or the CA3‐CA1 synapse. These results are further discussed below.

Previously, it has been proposed that hippocampal DSs occur in synchrony both longitudinally and between the two hemispheres (Bragin et al., 1995). Consistent with this we found that most of the DSs were simultaneous in the anterior and posterior dorsal hippocampus. This finding is explained by the fact that the perforant path spreads feed‐forward projections to all parts of the DG (Amaral & Witter, 1989; Vakilna et al., 2021). Surprisingly, contrary to previous reports (Bragin et al., 1995), we found that only a fraction of DSs were bilaterally synchronous. Based on anatomical evidence of EC‐EC and EC‐DG connections from one hemisphere to the other (Adelmann et al., 1996; Goldowitz et al., 1975), it seems possible that DSs could take place simultaneously in both hippocampi. However, the EC‐EC connections have been suggested to be di‐synaptic (Krug et al., 2001), and therefore there might be a delay of at least some 5–10 ms (one extra synapse) in the signal conductance from one EC to the contralateral DG, possibly partly explaining our finding. In addition, it has been demonstrated that EC projections to the contralateral DG are sparse compared to ipsilateral projections (Goldowitz et al., 1975), and that input from the EC to the contralateral DG is lower in amplitude. Thus, although the EC signal is relayed contralaterally, it is possible that the signal is not powerful enough to cause firing of granule cells and to induce a DS (Golarai & Sutula, 1996; Krug et al., 2001; Levy & Steward, 1979). It should also be noted that Urethane anesthesia as such is suggested to alter cortico‐hippocampal interactions (Headley et al., 2017) and to lower the excitability of the hippocampus (Dragunow et al., 1989; Riedel et al., 1994), yet it seems to modulate the brain functional connectivity only mildly (Paasonen et al., 2018).

In line with our findings, also others have reported evidence of functional lateralization of the rat hippocampus, especially regarding the CA3 (Benito et al., 2016; Jordan, 2020; Klur et al., 2009). A study in mice suggests that this may also be the case for the DG (Jordan et al., 2019). Further, recently it was reported that CA1 SPW‐Rs occur asynchronously between the hemispheres during slow‐wave sleep in rats (Villalobos et al., 2017). All in all, it seems plausible that DSs are mainly unilateral. The laterality of DSs (and SPW‐Rs) might also bear relevance to the function that they serve regarding learning and memory (Lensu et al., 2019; Nokia et al., 2017) and should be researched in the future in more detail.

As expected, when stimulating the PP during DSs, an increase in the synaptic response was evident in the DG. DSs seem to function as selective and phasic signal amplifiers between the EC and the DG. The effect was slightly more prominent after TBS, which is thought to mimic natural synaptic input to the DG when a rat is exploring its environment. That is, potentiation of the synapse (Bliss & Lømo, 1973) and increased excitability of the granule cells seemed to accentuate the impact of DSs on signal transmission from the EC to the DG. Possibly the role of increased granule cell excitability is to enhance plastic changes in the DG‐CA3 synapse (Bramham, 1998). PP is known to provide monosynaptic excitatory input also to mossy cells and hilar interneurons (Bragin et al., 1995; Penttonen et al., 1997; Sik et al., 1997), and although some of the mossy cells might be silent during DSs (Sanchez‐Aguilera et al., 2021), the enhanced response in the DG during DSs originates likely from the firing of all these DG cell types.

In our current study, the facilitating effect of DSs on synaptic transmission from the EC to the DG was present only during DSs and disappeared soon after them (>31 ms delay), whereas in an earlier study by Bramham (1998) the effect lasted for up to 100 ms. This difference might be explained by the fact that in Bramham's (1998) study the rats were awake whereas in the current study the rats were anesthetized, that is, the behavioral state was fixed. As mentioned earlier, urethane anesthesia has been suggested to alter the excitability of the hippocampus. Further, in our current study we used lower stimulation intensities (≤160 vs. ≤600 μA) than Bramham (1998), possibly causing lower excitation of granule cells. It should also be noted that the chronic implantations used by Bramham (1998) have been indicated to increase the excitability of DG granule cells (Löscher et al., 1995; Ruethrich et al., 1996). Regardless, in both these studies, the immediate facilitating effect of DSs on EC‐DG synaptic transmission was detected.

DSs are reported to have a suppressing effect on the excitability of the CA3 and CA1 pyramidal cells (Bragin et al., 1995; Penttonen et al., 1997). In our current experiment, DS‐contingent stimulation did not produce signal amplification nor attenuation in the DG‐CA3 or the CA3‐CA1 synapses. Thus, although the firing of principal cells in the CA3 and CA1 is suppressed during DSs, the excitability of the pyramidal cells seems to remain constant. This may indicate that instead of DSs leading to hyperpolarization or increased inhibition of the CA3‐CA1 pyramidal cells, there might be an interruption in the excitatory signal transduction from the DG to the CA3 pyramidal cells, and from the CA3 to the CA1 pyramidal cells. In addition, the propagation of signal directly from the EC to the CA1 also likely contributes to the net effect. The possible explanations for the cease fire in the CA3 and CA1 during DSs should be addressed in future studies. Although the duration of DSs is constant (~20 ms), there seems to be some variation in their amplitude, and in the future, it would be interesting to study how this variation influences synaptic responses in the hippocampus. For example, Headley et al. (2017) have indicated that DSs preceding SPW‐Rs are of lower amplitude than other DSs, such as those occurring after SPW‐Rs.

In conclusion, we demonstrated that the modulatory effect of DSs on signal transmission is temporally limited and only occurs in the EC‐DG synapse. The DS‐contingent silencing of CA1/3 pyramidal cells seems to be a result of a decrease in excitatory input rather than a decrease in the excitability of the pyramidal cells themselves. In addition, our findings provide novel evidence of the lateralization of the rodent hippocampus, as we found asynchronous DSs in the left and right hippocampi.

## FUNDING INFORMATION

This work was funded by the Academy of Finland (grant no. 316966 to MP and grant no. 321522 to MSN).

## CONFLICT OF INTEREST

None.

## Data Availability

Data available on request from the authors: The data that support the findings of this study are available from the corresponding author upon reasonable request.
